# Supplementary material for: Measurement and models accounting for cell death capture hidden variation in compound response
Source: Cell Death Dis. 2020 Apr 20;11(4):255. doi: 10.1038/s41419-020-2462-8 (PMC7171175; doi:10.1038/s41419-020-2462-8)
Supplement: Supplementary file 1 — Supplementary Figure Legends [file 41419_2020_2462_MOESM1_ESM.docx]

## Supplementary Figure Legends

**Figure S1:** **Endpoint measurements provide qualitatively similar results to those with kinetics.** Violin plots indicate the model posterior after fitting. While some difference exists in the growth and death rates for high doses of NVB, the overall trends are preserved with endpoint analysis. For example, NVB more potently induces cell death than DOX, and DOX is more biased toward driving apoptotic cell death. In fact, the fits to endpoint data seem more reasonable (e.g., decreased cell division in (d)), and so the increased growth rate with kinetic measurements may be an artifact of changes in cell response over the course of the experiment.

**Figure S2:** **Drug response measurements for a panel of targeted agents.** Model fits are shown in fig. [S3](#fig:S3), and results are summarized in fig. [3](#fig:targeted). Each line represents the mean of triplicate measurements for individual drug dose over time and shaded areas show the range of the measurements.

**Figure S3:** **Model fitting to the drug response panel in fig.**[**S2**](#fig:S2)**.** Violin plots indicate the model posterior after fitting to each treatment’s kinetic measurements.

**Figure S4:** **Drug response measurements for combinations of two agents with divergent phenotypic responses.** Drug response measurement of LCL161 (a) or PIM447 (b) combined with OSI-906. Each line represents the mean of triplicate measurements for individual drug dose over time and shaded areas show the ranges of measurements.
