# Supplementary figures and images for: Measurement and models accounting for cell death capture hidden variation in compound response
Source: Cell Death Dis. 2020 Apr 20;11(4):255. doi: 10.1038/s41419-020-2462-8 (PMC7171175; doi:10.1038/s41419-020-2462-8)

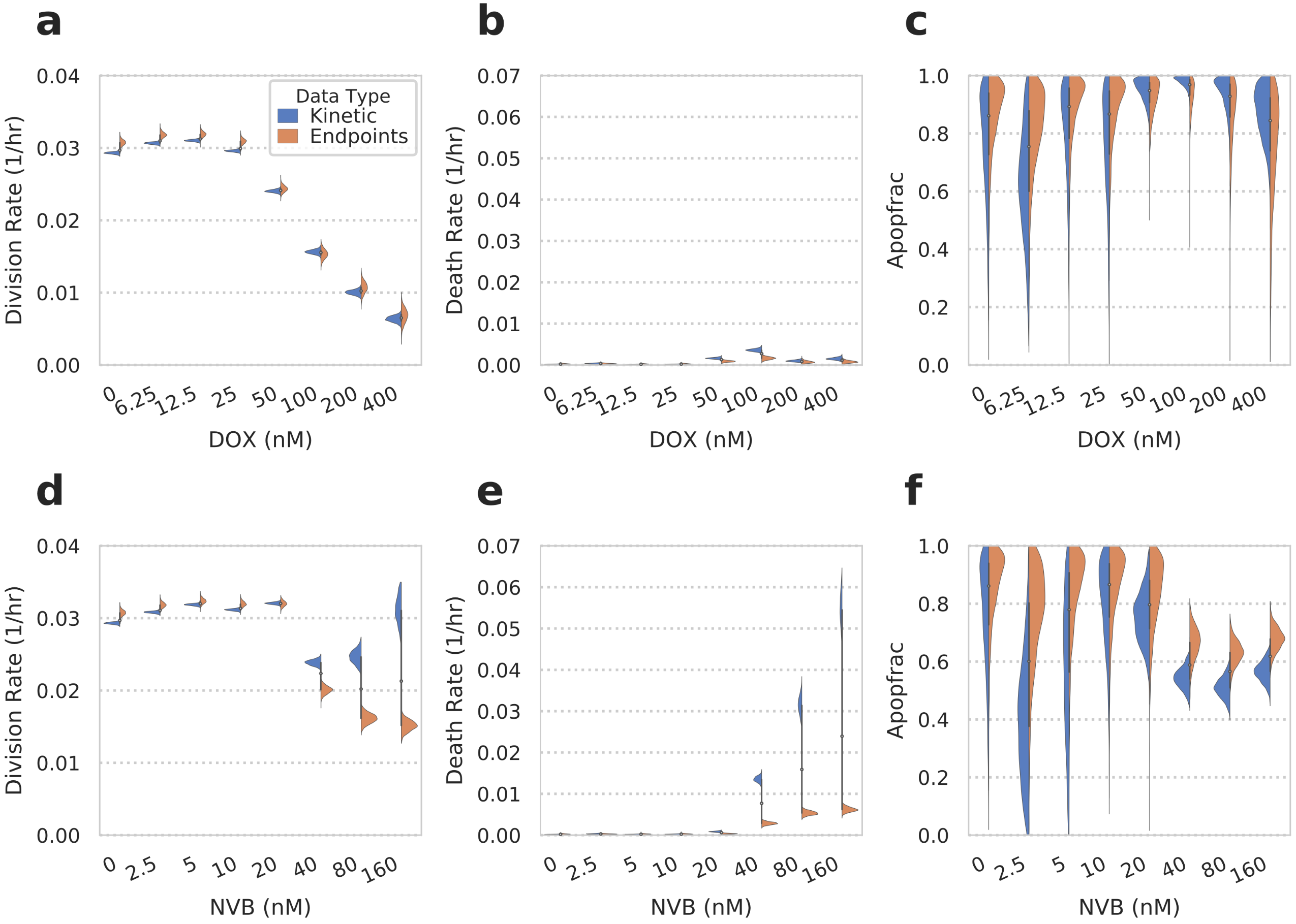

Supplement: Supplementary file 2 — Figure S1 [file 41419_2020_2462_MOESM2_ESM.png]

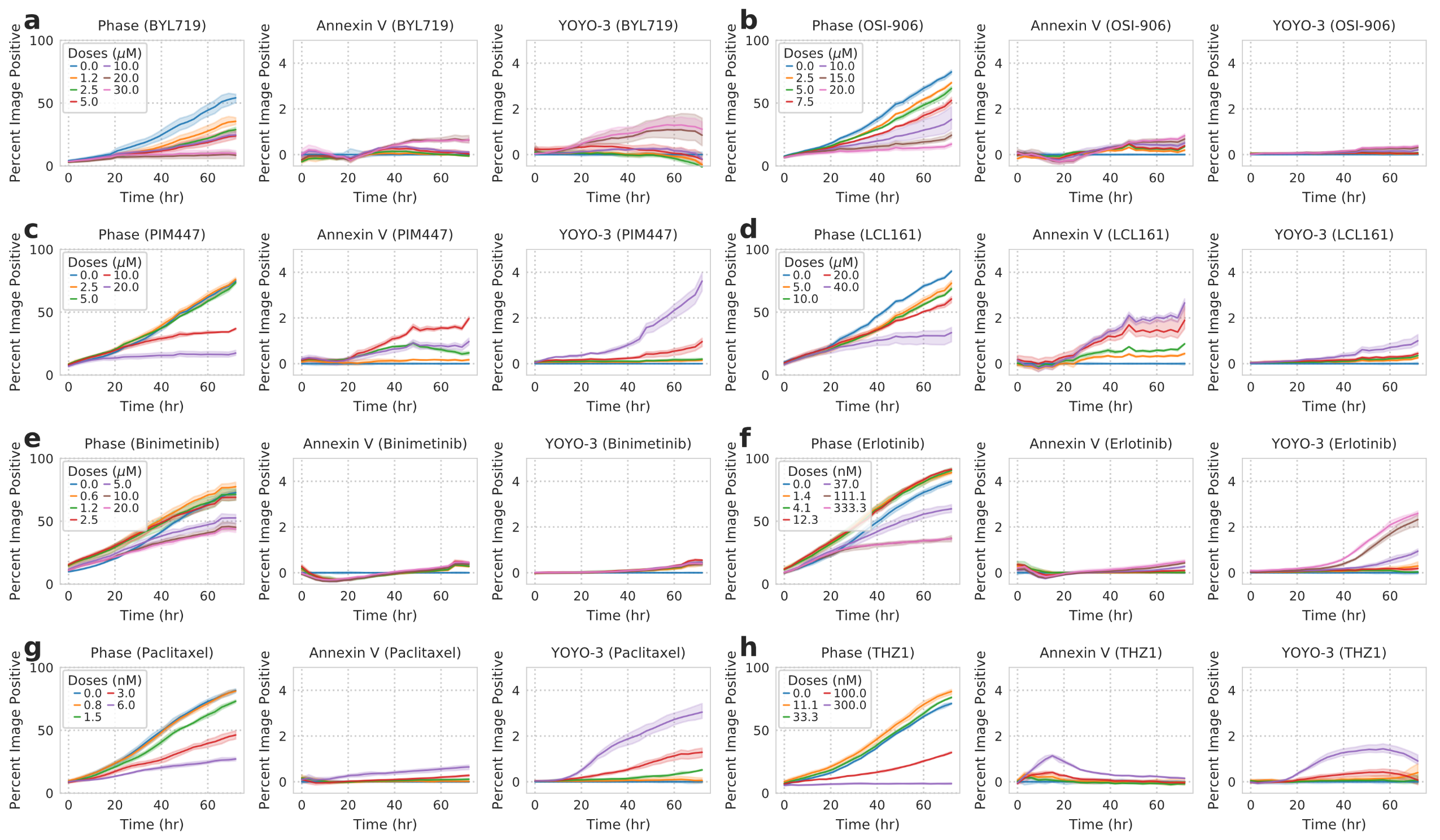

Supplement: Supplementary file 3 — Figure S2 [file 41419_2020_2462_MOESM3_ESM.png]

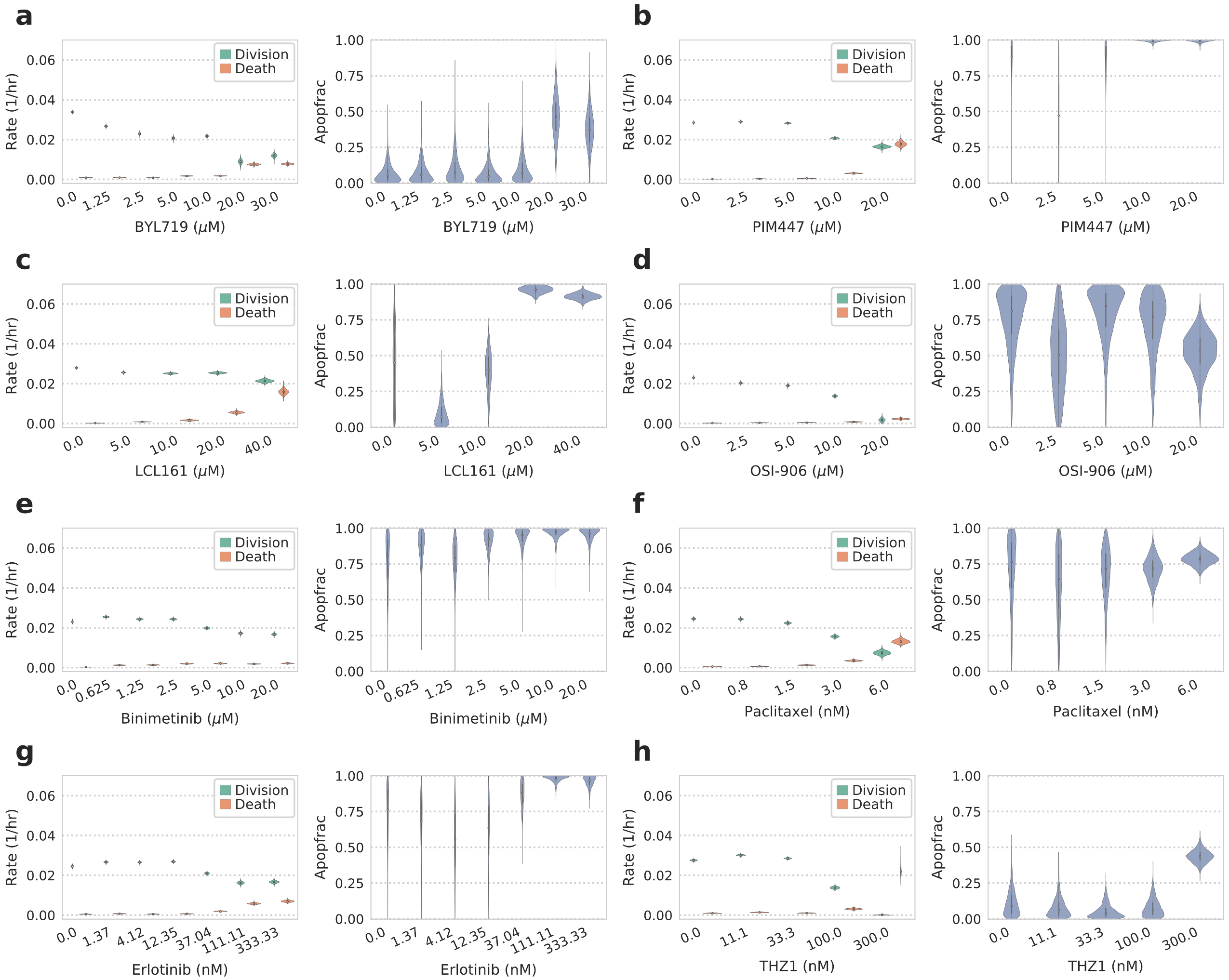

Supplement: Supplementary file 4 — Figure S3 [file 41419_2020_2462_MOESM4_ESM.png]

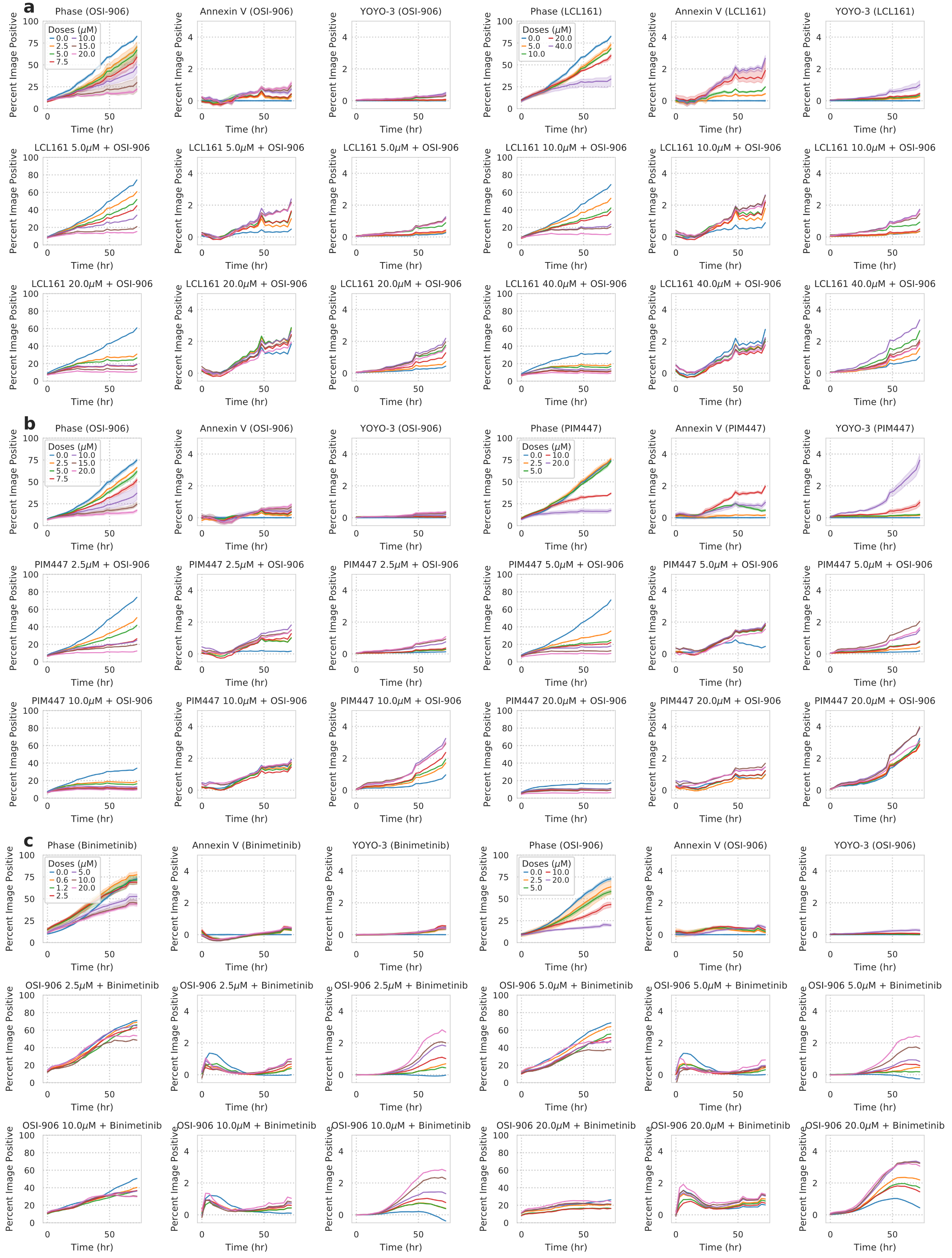

Supplement: Supplementary file 5 — Figure S4 [file 41419_2020_2462_MOESM5_ESM.png]
